# High MMP-9 Expression May Contribute to Retroprosthetic Membrane Formation after KPro Implantation in Rabbit Corneal Alkali Burn Model

**DOI:** 10.1155/2016/1094279

**Published:** 2016-02-01

**Authors:** Minghong Gao, Wei Sang, Fuying Liu, Hai Yu, Runhai Zhou, Michael Wellington Belin, Peter Zloty, Yingxin Chen

**Affiliations:** ^1^Department of Ophthalmology, General Hospital of Shenyang Military Area Command of Chinese PLA, Shenyang 110840, China; ^2^Dalian Medical University, Dalian 116044, China; ^3^Department of Ophthalmology, University of Arizona, Arizona Health Sciences Center, Tucson, AZ 85711, USA; ^4^Southeast Eye Clinic, Dothan, AL 36301, USA

## Abstract

*Purpose*. To evaluate aqueous humor MMP-9 levels in alkali-burned rabbit cornea following KPr implantation and their roles in RPMs formation.* Methods*. Left eyes of 36 rabbits received a deep corneal alkali wound. 12 corneas were implanted with KPro and the other 24 control corneas were either penetrating keratoplasty or left without keratoplasty. Aqueous humor MMP-9 and TIMP-1 levels were determined and RPMs were obtained for histopathological and ultrastructural examination.* Results*. Alkali exposure induced significant increase in aqueous humor MMP-9 level and the data were further enhanced by KPro implantation. By contrast, TMIP-1 levels in aqueous humor showed a decreased trend following corneal alkali burn and KPro surgery. RPMs were developed in 5 out of 10 cases of KPro successfully implanted eyes. Histopathology showed the presence of a large number of fibroblasts and collagen fibers arranged irregularly with inflammatory cells infiltration, and an ingrowth of new blood vessels in this retrokeratoprosthesis fibrous tissue. Immunohistochemical analysis showed positive stain of RPMs for both MMP-9 and TIMP-1. Aqueous humor MMP-9 levels were significantly higher in RPM group postoperatively, while TIMP-1 levels were comparatively lower than that of No-RPM group.* Conclusions*. Our study evidenced the potential pathophysiological role of MMP-9 expression in RPM formation following KPro implantation.

## 1. Introduction

Damage to the cornea from chemical burns is a serious clinical problem, accounting for about 7%–10% of eye injuries, of which, alkali-induced injury to cornea is the most dangerous, due to its quick penetration to the eye surface [1]. It can cause corneal infection, ulceration, perforation, neovascularization, and opacification [2] and often leads to permanent visual impairment. Penetrating keratoplasty has been used successfully to treat many corneal diseases; however, several intractable ocular surface diseases, such as chemical burns, are still untreatable using this procedure [3]. The Boston Type 1 keratoprosthesis (KPro), developed at the Massachusetts Eye and Ear Infirmary and approved by the US Food and Drug Administration (1993), is currently the most commonly used keratoprosthesis worldwide [4] and is used in cases at high risk for penetrating keratoplasty failure [5]. It has been successfully used to restore vision in cases of chronic corneal disease and graft failure, including corneal alkali burn [6]. However, despite multiple advances and improvements in prognosis of patients following KPro surgery, several challenges remain. The postoperative development of RPMs is one of the most common complications of keratoprosthesis surgery, affecting between 25% and 65% of patients, and is poorly understood [7, 8].

Matrix metalloproteinases (MMPs), zinc endopeptidase proenzymes responsible for the degradation of extracellular matrix components, play a role in many ocular physiological processes, including embryogenesis, angiogenesis, and wound healing [9]. Breakdown of the basement membrane by MMPs is known to contribute to the pathogenic ulceration and perforation of the corneal stroma [10]. The activity of MMPs is closely controlled by a family of natural antagonists, the tissue inhibitors of matrix metalloproteinases (TIMPs), whether MMPs exert normal or abnormal responses depends upon the balance between MMPs and TIMPs. An imbalance between MMPs and TIMPs in favor of inhibitor might provoke the development of fibrosis and in consequence may lead to tissue remodeling [11]. MMP-9 is the enzyme of major importance in the remodeling and degradation of the corneal stromal collagen in both animals and human beings [12]. It was found to be only expressed in rats which underwent corneal injury, instead of the normal corneal stroma [13]. The expression of MMP-9 and its endogenous inhibitor TIMP-1 in the aqueous humor of alkali-burned rabbit cornea before and after KPro implantation and their role in RPMs formation following KPro surgery have previously received limited attention. In this study, we report in greater detail the MMP-9 and TIMP-1 expressions in aqueous humor before and after KPro implantation into the alkali-burned rabbit cornea, the histopathological characteristics of RPMs developed after KPro implantation, and immunohistochemical expression of MMP-9 and TIMP-1 in this RPM.

## 2. Material and Methods

### 2.1. Animals

A total of 60 healthy adult New Zealand White rabbits, weighing between 2.5 and 3.0 kg, were obtained from the Animal Experimental Center of Army General Hospital of Shenyang Military Region (Shenyang, China). This study was approved by the Ethics Committee of Shenyang Military Region General Hospital. All experiments were performed on the left eye of animals and all animals and experimental conditions followed laboratory animal regulations of State Science and Technology Commission.

### 2.2. Experimental Model of Corneal Alkali Burn

Corneal alkali burn model was established as previously described with some modification [14]. To create an alkali burn, 50 rabbits were randomly selected and their left eyes were locally anesthetized with 0.5% Alcaine eye drops (Alcon Ophthalmic Products Co. Ltd., Shanghai, China). Then, a 10 mm in diameter Cellulose nitrate filter paper soaked in 1 mol/L NaOH was applied to the cornea for 45 s, followed by a rinse with 10 mL of balanced salt solution. After the corneal alkali burn was created, animals received chlortetracycline eye ointment (Beijing Shuangji Pharmaceutical Co., Ltd., China) and atropine sulphate eye gel (Shenyang Xingqi Pharmaceutical Co., Ltd., China) daily for one week.

The severity of conjunctival and corneal injury was determined according to the Roper-Hall (RHG) grading system [15]. Experimental model of corneal alkali burn was successfully established in 42 New Zealand White rabbits, as demonstrated by the presence of complete corneal opacity, corneal neovascularisation involving more than two quadrants of the cornea, and normal anterior chamber 3 months after severe alkali burn. The 36 model rabbits were randomly selected and divided into three groups (*n* = 12): group I, blank control group, corneal alkali burn without any keratoplasty; group II, control group, cornea alkali burn with penetrating keratoplasty; group III, experimental group, corneal alkali burn with KPro implantation. The 10 untouched rabbits served as allogeneic cornea donors after collection of aqueous humor samples.

### 2.3. Corneal Implantation

The surgery was performed as previously described with some modification [16]. The procedures were performed under a surgical microscope (Zeiss, Jena, Germany) by a single investigator (Yinxin Chen), and the surgical time was 45 to 60 min per procedure. Briefly, donor cornea was marked in the periphery with an 8.5 mm diameter biopsy punch (Miltex, Plainsboro, NJ, USA) and then excised with Vannas scissors (Storz Instruments Company, San Damis, CA, USA) after animal euthanasia.

The corneas were trephined centrally with a 3 mm diameter punch, slid over the stem of the PMMA front plate, followed by titanium back plate position (KPro device, 8.5 mm in diameter, Boston Keratoprosthesis Operations, MA, USA), and complex was then placed in PBS. Three months after injury, the recipient cornea was marked with an 8.5 mm diameter biopsy punch and excised with Vannas scissors under general and topical anesthesia (120 mg/kg ketamine and 20 mg/kg xylazine). The extracapsular crystalline lens extraction was then performed on eyes of groups II and III and the rabbits were left aphakic with an intact posterior capsule. The lenses of group I eyes were not extracted and the rabbits were left phakic. The donor cornea KPro complex was then positioned in the recipient bed and secured with sixteen interrupted 11.0 nylon sutures (Sharpoint; Angiotech Pharmaceuticals, Vancouver, BC, Canada). A drop of tobramycin and dexamethasone (Tobradex; Alcon, Puurs, Belgium) was applied and a tarsorrhaphy was performed using 8-0 nylon sutures. The tarsorrhaphy was removed 48 h after the procedure, and the same antibiotic and steroid drop was administered once a day for 7 consecutive days following tarsorrhaphy removal.

After operation, animals received tobramycin and dexamethasone ophthalmic ointment once daily for three days, atropine sulfate eye gel once daily for one week, chloramphenicol eye drops four times daily for 4 weeks, and chlortetracycline hydrochloride eye ointment once every night for 4 weeks.

### 2.4. Follow-Up and Clinical Evaluation

RPM referred to membrane growing on the back of the device, all eyes were determined under slit-lamp, and RPMs were diagnosed at the time of explantation. The aqueous humor was obtained by puncturing the anterior chamber using a 45 gauge needle at postoperative days 7, 14, 28, 60, and 90. MMP-9 and TIMP-1 levels in aqueous humor were determined using the commercially available ELISA kits for MMP-9 and TIMP (Shanghai Sunred Biological Technology Co., Ltd., China). Ninety days after implantation, all rabbits were sacrificed by using the air embolism method by injecting 20 mL of air into the ear marginal vein. The presence of complications, like retinal detachment, intraocular infection, and so forth, was assessed by B ultrasound. The RPMs that developed were then excised and histological sections were prepared for further processing.

Slit-lamp examination was performed before surgery and at postoperative days 7, 14, 28, 60, and 90, to examine the corneas for signs of inflammation or neovascularization and to assess optical transparency, and photographs were taken. For transmission electron microscopy (TEM), the sections were stained with uranium acetate and lead citrate and observed with JEM-1200 transmission electron microscopy (Tokyo, Japan). Some pieces were fixed in 4% paraformaldehyde in 0.1 mol/L PBS, paraffin embedded, and then sectioned. Hematoxylin and eosin (H&E) staining was performed for routine histopathologic examination. The immunohistochemical staining was performed using rat anti-rabbit polyclonal antibodies against MMP-9 and TIMP-1 and biotin-labeled sheep anti-mouse IgG, all purchased from Beijing Boaosen Biotechnology Co., Ltd., China. Five sections were randomly selected and data were obtained from 50 fields of view (10 fields from each section).

### 2.5. Statistical Analysis

Data were expressed as mean ± SD and analyzed using SPSS 19.0 for windows (SPSS Inc., Chicago, Illinois, USA). The One-Way ANOVA procedure was used to determine the difference among groups. A *p* value of <0.05 was considered statistically significant.

## 3. Results

### 3.1. Clinical Results

**Figure 1:**
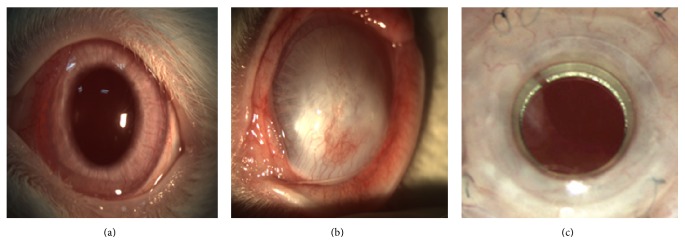
Slit-lamp appearance of anterior segment of the rabbit eyes before alkali burn (a), three months after alkali burn (b), and after implantation of Boston Type 1 keratoprosthesis (KPro), with the retroprosthetic membranes developed (c).

**Figure 2:**
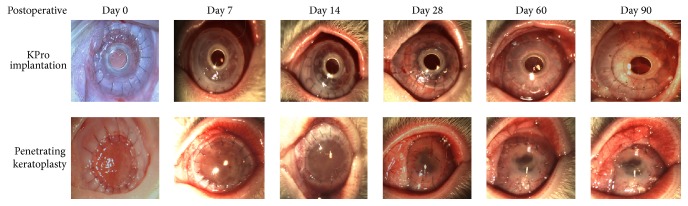
Clinical observation of alkali-burned rabbit corneas following Boston Type 1 keratoprosthesis (KPro) implantation or penetrating keratoplasty. Photographs taken immediately after operation and at postoperative days 7, 14, 28, 60, and 90.

Three months after the infliction of alkali wounds, the injured corneas exhibited stromal opacities of varying depth; some corneas completely became opaque, when compared with the normal ones (Figures 1(a) and 1(b)). Slit-lamp examination showed RPMs developed 30 ± 10 days after transplantation of KPro (Figure 1(c)). After corneal transplantation, 1 rabbit accidently died in group II and 2 in group III. The common symptom in early period of postoperation were eyelid margin redness and swelling, conjunctival injection, tearing, photaesthesia and white secretion from the conjunctival sac. The local irritation lasted between 7 and 14 days in group II and between 7 and 21 days in group III. There was a clear boundary of edema on the cornea graft; then the edema reduced gradually at postoperative day 7 and the corneal grafts became clear (Figure 2). At the postoperative day 14, there was new vessel growth in the upper corneal limbus, and a large amount of new vessels formed 28 days postoperatively (Figure 2). At 60 and 90 postoperative days, a reduction in the number of vessels and level of haze was noted relative to earlier examinations (Figure 2). The postoperative complication in group III included RPMs in 5 eyes (Figure 2, day 14) and vitreous hemorrhage in two eyes. However, in group II, corneal rejection occurred in 5 eyes 14 days after penetrating keratoplasty, with diffuse edema and opacity of the graft (Figure 2). The shallow anterior chamber and anterior synechiae developed in 1 case and posterior capsule opacification developed in 3 cases (Figure 2, day 60). There were no postoperative retinal detachment and endophthalmitis in the both groups II and III.

### 3.2. Aqueous Humour MMP-9 and TIMP-1 Levels following KPro Implantation

The dynamic expressions of MMP-9 and TIMP-1 in aqueous humour were quantified using commercially available ELISA kit; our results showed that there was a small amount of MMP-9 and TIMP-1 in normal rabbit aqueous humor (12.52 ± 3.05 and 33.49 ± 3.39 ng/mL, resp.). The severe alkali burn employed in this study resulted in a significant increase in both MMP-9 and TIMP-1 levels three months later.

After corneal transplantation, aqueous humor MMP-9 in group III showed an increasing trend and peaked at postoperative day 90. By contrast, in group II, MMP-9 in aqueous humour reached peak at postoperative day 14 and then significantly decreased thereafter, although the data at postoperative day 90 was still significantly higher than that of preoperative level and of group I (*p* < 0.05, Table 1).

However, unlike the increase in MMP-9 postoperatively, aqueous humor TIMP-1 was significantly decreased following operation in both groups II and III. The data reached valley bottom at postoperative day 90 in group III and at postoperative day 14 in group II and then increased until the postoperative day 90 (*p* < 0.05 versus preoperative level and blank control, Table 1).

The concentration of MMP-9 in group III became higher on the postoperative day 28 when compared with group II, and the difference was statically significant at subsequent points (*p* < 0.05). By contrast, TIMP-1 level became lower on the postoperative day 28 and the data became statistically significant thereafter when compared with group II (*p* < 0.05).

### 3.3. Histopathological and Immunohistochemical Analysis of Retroprosthetic Membrane Specimens

**Figure 3:**
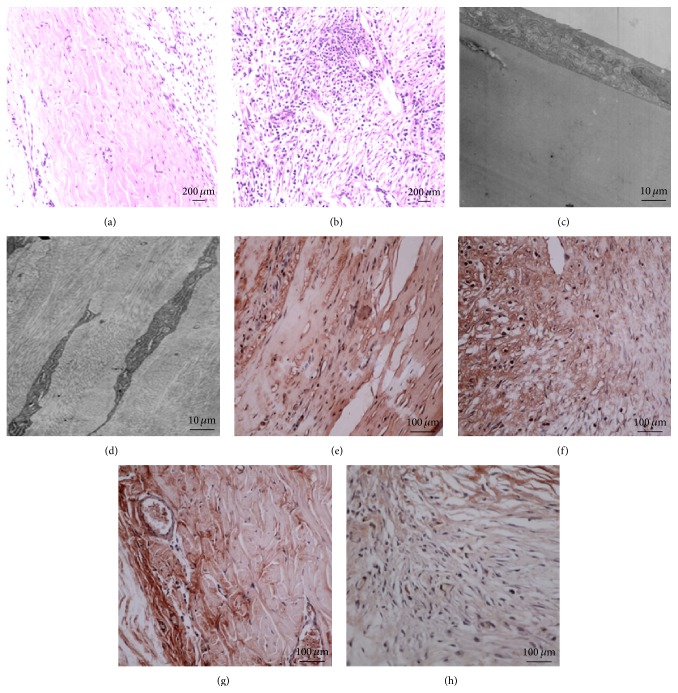
Histopathology of retroprosthetic membrane extracted from the explanted keratoprosthesis in the corneal alkali-burned rabbits. H&E staining of normal cornea ((a) ×100) and retroprosthetic membrane ((b) ×100); transmission electron microscopy of normal cornea ((c) ×4000) and fibrous retroprosthetic membrane ((d) ×4000); immunohistochemical analysis of MMP-9 ((e) ×200) and TIMP-1 ((f) ×200) expressions in normal cornea (MMP-9: (e) ×200; TIMP-1: (g) ×200) and retroprosthetic membrane (MMP-9: (f) ×200; TIMP-1: (h) ×200).

In total, five retroprosthetic membrane specimens were obtained following Boston KPro explantation; our hematoxylin-eosin staining showed a large number of fibroblasts and collagen fibers arranged irregularly and inflammatory cell infiltration. There was ingrowth of new blood vessels in the RPMs (Figures 3(a) and 3(b)). Transmission electron microscopy of the retro-KPro fibrous membrane revealed an abundance of elongated collagenous fibers, separated by fibroblasts and granulocyte infiltration. These cells were spindle-shaped with mitochondria and rough endoplasmic reticulum in the cytoplasm. The extracellular space separating these cells was composed of fibroblasts (Figures 3(c) and 3(d)). Immunohistochemical results showed positive staining of retroprosthetic membrane specimens for MMP-9 and TIMP-1, with yellowish-brown cytoplasm and blue nucleus (Figures 3(e), 3(f), 3(g), and 3(h)).

### 3.4. Aqueous Humor MMP-9 and TIMP-1 Levels in KPro-Implanted Rabbits with or without RPM

**Figure 4:**
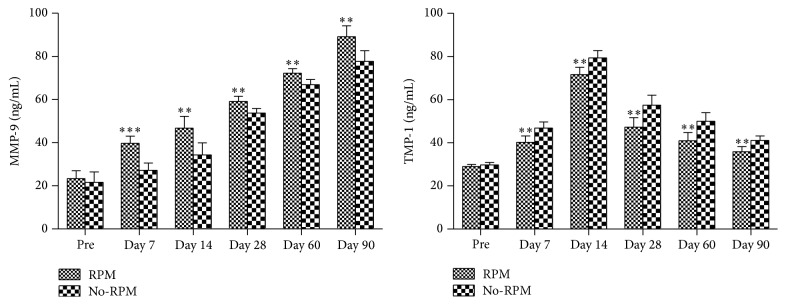
Aqueous humor levels of MMP-9 and TIMP-1 in Boston Type 1 keratoprosthesis (KPro) implanted rabbits with or without RPM formation. Pre: preoperation; Day: postoperative day; RPM: retroprosthetic membrane. ^*∗∗*^ *p* < 0.01, ^*∗∗∗*^ *p* < 0.001 versus the No-RPM group.

To further explore the role of MMP-9 and TIMP-1 expressions in retroprosthetic membrane formation following the KPro implantation, rabbits with KPro implantation were subdivided into RPM and No-RPM groups. Aqueous humor levels of MMP-9 were significantly higher in the RPM group postoperatively when compared with that in the No-RPM group (*p* < 0.01 or *p* < 0.001). By contrast, Aqueous humor TIMP-1 levels in the RPM group were comparatively lower than that of No-RPM group following the operation (*p* < 0.01). There was no significant difference between the two groups in preoperative aqueous humor MMP-9 and TIMP-1 levels (Figure 4).

## 4. Discussion

Ocular trauma in the form of an alkali burn to the cornea is among the most serious ocular injuries that may cause severe and permanent visual impairment [17]. It can induce a strong inflammatory reaction characterized by cell infiltration and production of proteolytic enzymes and cytokines [1]. Previous studies have indicated an indispensable role for MMPs in the homeostasis and pathophysiology of the cornea [18]. MMPs and TIMPs are present in the aqueous humor [19], and alkali injury enhanced the expression of MMP-9 in mice [20]. Degradation of the basement membrane by proteolytic enzyme MMP-9 may involve the pathogenic process of alkali injury [21, 22]. The presence of MMP-9 has been identified in the tear fluid of patients with corneal alkali burn [23]. Consistent with the results of this study, our results showed that alkali insult of rabbit cornea resulted in a significant increase in aqueous humor MMP-9 levels, while the TIMP-1 levels in aqueous humor were found to decrease to some extent three months after corneal alkali burns. After keratoplasty, MMP-9 levels in aqueous humor were further significantly enhanced in experimental rabbits within the postoperative day 90, whereas TIMP-1 levels were downregulated further when compared with that in group I (blank control). All these results showed that evidence suggested the pathophysiological role of MMP-9 in alkali-induced corneal injury and repair.

The Boston keratoprosthesis is by far the most commonly used prosthetic cornea in the world [24]. However, there are a number of significant complications that can occur postoperatively. Among which, retroprosthetic membrane is one of the most common postoperative complications encountered [25], occurring in 25% to 65% of patients within a year after KPro implantation, and up to 45% of cases require eventual treatment [26, 27]. The formation of retroprosthetic membrane was shown to correlate with the risk of corneal melt [28]. The etiology of retroprosthetic membrane is still largely unknown. However, the presence of this retrokeratoprosthesis fibrous tissue may act as a physical barrier between the cornea and the aqueous humor, which limits the contact between the aqueous and the corneal graft enough, and ultimately result in stromal necrosis and epithelial degeneration in KPro implanted eyes [29]. In our study, retroprosthetic membrane happened in 5 out of 10 KPro-implanted rabbits (50%), indicating the high incidence of retroprosthetic membrane formation following KPro implantation. Previous study showed that chemical injury seems to be an insignificant risk factor for the development of RPM [5], while other study also indicated that chemical burns have higher incidence of RPM development [30]. However, it is not possible to state whether alkali burn used in this series could be a contributing factor in the higher rate of RPM formation reported herein, since, to the best of our knowledge, there seems to be few researches on KPro implantation in animal model of corneal alkali burns up to now. The RPMs have been previously evaluated in patients with KPro implantation and were suggested to vary according to antecedent clinical events of the patients [7, 29]. Therefore, we further evaluated the histopathological characteristics of the RPMs obtained, as well as the immunohistochemical expression of MMP-9 and TIMP-1 in these RPMs. Our results showed a large number of fibroblasts and collagen fibers arranged irregularly with inflammatory cells infiltration, as well as ingrowth of new blood vessels in this retrokeratoprosthesis fibrous tissue. Corroborating the results of MMP-9 and TIMP-1 expressions in aqueous humor, our immunohistochemical analysis showed positive staining of retroprosthetic membrane specimens for MMP-9 and TIMP-1. Moreover, aqueous humor MMP-9 levels were found to be significantly higher in KPro-implanted eyes with RPM developed when compared with that of No-RPM developed ones. By contrast, aqueous humor TIMP-1 levels were comparatively lower than that of No-RPM group. All these results suggested that higher expression of MMP-9 may contribute to retroprosthetic membrane formation following KPro implantation.

There are several limitations in our study. The most important one is that the lens status was different among the black control group (group I), control group (group II), and KPro group (group III). Lens status has been reported to be a confounding factor on MMP-9 and TIMP-1 levels [31, 32], which may partially contribute to the elevated MMP-9 and decreased TIMP-1 levels observed in our study. However, despite the potential effect of lens status, our study did show the potential pathophysiological role of MMP-9 in RPMs formation, as demonstrated by significantly different aqueous humor MMP-9 and TIMP-1 levels between KPro-implanted eyes with and without RPM development.

In conclusion, the finding of our study showed the upregulated expression of MMP-9 and downregulated endogenous MMP-9 inhibitor, TIMP-1, after alkali-induced injury to the rabbit corneas and following KPro implantation, and to the best of our knowledge this study is the first to show the potential pathophysiological role of MMP-9 in RPMs formation following KPro implantation in a rabbit model of corneal alkali burn.

## Figures and Tables

**Table 1:** Dynamic expressions of MMP-9 and TIMP-1 in aqueous humor of alkali-burned rabbit cornea following Boston Type 1 keratoprosthesis (KPro) implantation.

Time	MMP-9 (ng/mL)			TIMP-1 (ng/mL)		
	Group III	Group II	Group I	Group III	Group II	Group I
Preoperation	22.85 ± 4.20	22.52 ± 4.03	22.39 ± 3.36	29.42 ± 1.05	29.44 ± 1.08	29.37 ± 1.01
Day 7	33.48 ± 7.29^*∗*#^	43.54 ± 4.39^#^	22.47 ± 4.13	27.44 ± 1.16^#^	26.57 ± 1.94^#^	29.46 ± 1.14
Day 14	40.55 ± 8.43^*∗*#^	75.48 ± 5.20^#^	22.41 ± 3.56	24.51 ± 1.98^*∗*#^	21.45 ± 3.59^#^	29.51 ± 1.31
Day 28	56.42 ± 3.56^#^	52.39 ± 6.83^#^	22.40 ± 4.15	21.44 ± 3.57^#^	23.50 ± 2.77^#^	29.52 ± 1.13
Day 60	69.55 ± 3.52^*∗*#^	45.44 ± 6.02^#^	22.39 ± 4.16	19.42 ± 1.96^*∗*#^	25.41 ± 1.96^#^	29.54 ± 1.30
Day 90	83.42 ± 7.63^*∗*#^	38.53 ± 3.54^#^	22.36 ± 3.55	14.49 ± 4.36^*∗*#^	28.46 ± 6.84	29.58 ± 1.37

Group III: corneal alkali-burned rabbits implanted with Boston Type 1 keratoprosthesis (KPro); group II: corneal alkali-burned rabbits with penetrating keratoplasty; group I: corneal alkali-burned rabbits without keratoplasty. ^*∗*^ *p* < 0.05 versus group II, ^#^ *p* < 0.05 versus group I.

## Abbreviations

### Abbreviations

MMP-9: Matrix metalloproteinases 9
TIMP-1: Tissue inhibitors of matrix metalloproteinase 1
KPro: Boston Type 1 keratoprosthesis
RPM: Retroprosthetic membrane.

## References

[1] He J., Bazan N. G., Bazan H. E. P. (2006). Alkali-induced corneal stromal melting prevention by a novel platelet-activating factor receptor antagonist. *Archives of Ophthalmology*.

[2] Salman İ. A., Gündoğdu C. (2010). Epithelial healing in experimental corneal alkali wounds with nondiluted autologous serum eye drops. *Cutaneous and Ocular Toxicology*.

[3] Kim M. K., Wee W. R., Lee J. H. (2015). Korea Seoul-type KPro: indications, contraindications, and surgical techniques. *Keratoprostheses and Artificial Corneas*.

[4] Robert M.-C., Dohlman C. H. (2014). A review of corneal melting after Boston keratoprosthesis. *Seminars in Ophthalmology*.

[5] Rudnisky C. J., Belin M. W., Todani A. (2012). Risk factors for the development of retroprosthetic membranes with Boston keratoprosthesis type 1: multicenter study results. *Ophthalmology*.

[6] Al Arfaj K., Hantera M. (2012). Short-term visual outcomes of Boston keratoprosthesis type I in Saudi Arabia. *Middle East African Journal of Ophthalmology*.

[7] Stacy R. C., Jakobiec F. A., Michaud N. A., Dohlman C. H., Colby K. A. (2011). Characterization of retrokeratoprosthetic membranes in the Boston type 1 keratoprosthesis. *Archives of Ophthalmology*.

[8] Hou J. H., Sivaraman K. R., De La Cruz J., Lin A. Y., Cortina M. S. (2014). Histopathological and immunohistochemical analysis of melt-associated retroprosthetic membranes in the Boston type 1 keratoprosthesis. *JAMA Ophthalmology*.

[9] Kawashima Y., Saika S., Miyamoto T. (2000). Matrix metalloproteinases and tissue inhibitors of metalloproteinases of fibrous humans lens capsules with intraocular lenses. *Current Eye Research*.

[10] Daniels J. T., Geerling G., Alexander R. A., Murphy G., Khaw P. T., Saarialho-Kere U. (2003). Temporal and spatial expression of matrix metalloproteinases during wound healing of human corneal tissue. *Experimental Eye Research*.

[11] Kraus M., Filarska M. K., Tomkowicz A. (2007). Metalloproteinases and airway remodeling in asthma. *Advances in Clinical and Experimental Medicine*.

[12] Trujillo Piso D. Y., Ribeiro A. P., Silva M. L. (2014). Effects of antiproteolytic agents on corneal epithelial viability and matrix metalloproteinase-2 and metalloproteinase-9 activity in alkali-burned corneas of rats. *Veterinary Ophthalmology*.

[13] Matsubara M., Girard M. T., Kublin C. L., Cintron C., Fini M. E. (1991). Differential roles for two gelatinolytic enzymes of the matrix metalloproteinase family in the remodelling cornea. *Developmental Biology*.

[14] Ye J., Yao K., Kim J. C. (2006). Mesenchymal stem cell transplantation in a rabbit corneal alkali burn model: engraftment and involvement in wound healing. *Eye*.

[15] Roper-Hall T., Eagling E. (1986). Burns to the eye and periorbital tissue. *Eye Injuries*.

[16] Crnej A., Omoto M., Dohlman T. H. (2014). A novel murine model for keratoprosthesis. *Investigative Ophthalmology and Visual Science*.

[17] Brodovsky S. C., McCarty C. A., Snibson G. (2000). Management of alkali burns: an 11-year retrospective review. *Ophthalmology*.

[18] Fini M. E., Parks W. C., Rinehart W. B. (1996). Role of matrix metalloproteinases in failure to re-epithelialize after corneal injury. *The American Journal of Pathology*.

[19] Di Girolamo N., Verma M. J., McCluskey P. J., Lloyd A., Wakefield D. (1996). Increased matrix metalloproteinases in the aqueous humor of patients and experimental animals with uveitis. *Current Eye Research*.

[20] Lu P., Li L., Liu G., Rooijen N. V., Mukaida N., Zhang X. (2009). Opposite roles of CCR2 and CX3CR1 macrophages in alkali-induced corneal neovascularization. *Cornea*.

[21] Saika S., Yamanaka O., Okada Y. (2007). Effect of overexpression of ppar*γ* on the healing process of corneal alkali burn in mice. *The American Journal of Physiology—Cell Physiology*.

[22] Kim E. C., Kim T. K., Park S. H., Kim M. S. (2012). The wound healing effects of vitamin A eye drops after a corneal alkali burn in rats. *Acta Ophthalmologica*.

[23] Sakimoto T., Sawa M. (2012). Metalloproteinases in corneal diseases: degradation and processing. *Cornea*.

[24] Ament J. D., Stryjewski T. P., Ciolino J. B., Todani A., Chodosh J., Dohlman C. H. (2010). Cost-effectiveness of the Boston keratoprosthesis. *American Journal of Ophthalmology*.

[25] Dunlap K., Chak G., Aquavella J. V., Myrowitz E., Utine C. A., Akpek E. (2010). Short-term visual outcomes of boston type 1 keratoprosthesis implantation. *Ophthalmology*.

[26] Bradley J. C., Hernandez E. G., Schwab I. R., Mannis M. J. (2009). Boston type 1 keratoprosthesis: the University of California Davis experience. *Cornea*.

[27] Aldave A. J., Kamal K. M., Vo R. C., Yu F. (2009). The boston type I keratoprosthesis: improving outcomes and expanding indications. *Ophthalmology*.

[28] Sivaraman K. R., Hou J. H., Allemann N., de la Cruz J., Cortina M. S. (2013). Retroprosthetic membrane and risk of sterile keratolysis in patients with type I Boston keratoprosthesis. *American Journal of Ophthalmology*.

[29] Güell J. L., Arrondo E., Cortina M. S. (2015). Boston KPro type I: complications. *Keratoprostheses and Artificial Corneas*.

[30] Todani A., Ciolino J. B., Ament J. D. (2011). Titanium back plate for a PMMA keratoprosthesis: clinical outcomes. *Graefe's Archive for Clinical and Experimental Ophthalmology*.

[31] Pollreisz A., Sacu S., Eibenberger K. (2015). Extent of detached retina and lens status influence intravitreal protein expression in rhegmatogenous retinal detachment. *Investigative Opthalmology & Visual Science*.

[32] Colitz C. M. H., Saville W. J. A., Renner M. S. (2010). Risk factors associated with cataracts and lens luxations in captive pinnipeds in the United States and the Bahamas. *Journal of the American Veterinary Medical Association*.

